# 3M syndrome in Saudi Arabia: a case series study and literature review

**DOI:** 10.3389/fendo.2025.1666468

**Published:** 2025-11-11

**Authors:** Raghad Alhuthil, Afaf Alsagheir, Maha Almslam, Jana Raed, Farah Barakat, Sarah Murad, Bassam Bin-Abbas

**Affiliations:** 1 Department of Pediatrics, King Faisal Specialist Hospital & Research Centre, Riyadh, Saudi Arabia; 2 College of Medicine, Alfaisal University, Riyadh, Saudi Arabia; 3 Department of Radiology, King Faisal Specialist Hospital and Research Centre, Jeddah, Saudi Arabia

**Keywords:** 3M syndrome, growth hormone therapy, rare hereditary disorder, CUL7, OBSL1, CCDC8

## Abstract

**Background:**

3M syndrome (3MS) is a very rare autosomal recessive disorder characterized by short stature, distinctive facial features, and skeletal abnormalities. The condition is frequently underdiagnosed due to its nonspecific symptoms and normal neurocognitive development. Few reports exist on its clinical course and response to growth hormone (GH) therapy. Therefore, this study aims to describe the clinical features of Saudi patients with 3MS and to investigate the effects of growth hormone therapy on growth.

**Methods:**

We conducted a retrospective case series of 14 Saudi patients from 11 families with genetically confirmed 3MS at King Faisal Specialist Hospital and Research Centre in Riyadh.

**Results:**

The mean age at diagnosis was 5.4 years. Consanguinity was present in 79% of cases. The most frequently affected gene was *CUL7* (57% of cases), followed by *OBSL1* and *CCDC8*. All variants were predominantly homozygous and classified as pathogenic or likely pathogenic. Clinical abnormalities included growth retardation, dental abnormalities, spinal abnormalities, and a characteristic facial appearance. GH therapy was administered to 10 children; 5 demonstrated a measurable improvement in growth velocity, while 5 did not respond or discontinued treatment. IGF-1 was within/low-normal in most tested cases, with two elevated results.

**Conclusion:**

Our study highlights the extensive phenotypic variability of 3MS and underscores the predominantly autosomal recessive inheritance pattern in this population. GH therapy may provide a growth benefit in select cases, although resistance and poor response remain a challenge. Genetic testing is crucial for accurate diagnosis, individualized management, and appropriate family counseling.

## 1 Introduction

3M syndrome (3MS) is a rare autosomal recessive disorder characterized by short stature, distinctive facial features, and skeletal abnormalities (1). It is inherited in accordance with an autosomal recessive pattern (1), and is considered very uncommon, with approximately 200 cases reported worldwide. The actual prevalence, however, might be higher because many cases might go unnoticed due to normal cognitive development (2, 3).

Individuals diagnosed with 3MS experience profound prenatal growth retardation, attributed to fetal growth delays, leading to a diminished birth weight (1). The growth impediments persist beyond birth, manifesting as a consistent pattern of delayed growth throughout childhood and adolescence, culminating in a stature significantly below the average. Many distinctive physical characteristics associated with this condition are congenital in nature. Craniofacial anomalies commonly observed encompass a disproportionately elongated and narrow head, a prominently broad forehead, and a triangular facial appearance marked by a hypoplastic midface, pointed chin, extended philtrum, noticeable mouth, depressed nasal bridge, fleshy-tipped upturned nose, large ears, and full lips (1, 4–6).

While skeletal anomalies are not apparent at birth, they gradually manifest, including delayed bone maturation, elongated and slender tubular bones, and heightened vertebral bodies (1, 4). Some individuals exhibit joint hypermobility and an increased susceptibility to hip dislocation (1, 5). Anomalous spinal curvature, such as kyphoscoliosis or hyperlordosis, leading to back pain, is also documented in this disorder (1, 5).

Additional physical abnormalities identified in certain children consist of an unusually short and broad neck and thorax, square shoulders, flared shoulder blades, atypical curvature of the 5th finger, and prominent heels (1, 5, 6).

Three different genes have been involved in the disease so far, with mutations in *CUL7*, *OBSL1* and *CCDC8* (1). The *CUL7* gene, initially documented in 2005 (7), is responsible for 77.5% of genetically confirmed cases, with a specific mutation identified in exon 24 for Maghreb families (7–9).

Consequently, there is limited data on this syndrome in the existing literature. Therefore, the purpose of this study is to describe the clinical characteristics of 14 Saudi patients who have 3MS and investigate the impact of growth hormone (GH) therapy on their growth.

## 2 Methodology

This retrospective case series research involved 14 cases of 3MS who are currently receiving care at endocrinology clinics at King Faisal Specialist Hospital and Research Centre (KFSHRC) in Riyadh, Saudi Arabia. Data retrieval occurred from November 2023 to January 2025 from our database and included both pediatric and adult patients. Individuals without available genetic testing data were excluded. The study documented patients’ demographics, medical history, presentations, management and investigative results. Approval for this study was obtained from the Office of Research Affairs at King Faisal Specialist Hospital and Research Centre (reference number: 2245444).

The diagnostic criteria for 3MS include proportionate short stature, characteristic facial and skeletal features, with confirmation typically achieved by identifying pathogenic variants in the *CUL7*, *OBSL1*, or *CCDC8* genes.

Genetic testing was conducted as part of routine clinical practice. Following patient consent, DNA was extracted from peripheral blood samples, and whole-exome sequencing was carried out at the Molecular Diagnostic Laboratory of the Clinical Genomic Department, Center for Genomic Medicine at KFSHRC.

Clinical improvement with GH therapy is defined as a significant and sustained increase in height velocity (≥2 cm/year above baseline) and/or an improvement in height SDS (≥0.3–0.5 within one year), without adverse effects, indicating partial restoration of growth potential.

This research was performed according to the guidelines of the Declaration of Helsinki and approved by the Office of Research Affairs in King Faisal Specialist Hospital and Research Centre (Reference number: 2231134). This was a retrospective study; therefore, informed consent was not required.

## 3 Results

**Table 1** presents clinical and genetic characteristics of 14 individuals (10 females, 4 males) from 11 families diagnosed with 3MS. The mean current age of the cases was approximately 11.2 years, with a range of 4 to 39 years. The average age at diagnosis (excluding intrauterine cases) was 5.4 years. The most commonly affected gene was *CUL7* (8/14 cases), followed by *OBSL1* (5/14), and *CCDC8* (2/14). One subject had no gene documented. Most variants were homozygous (13/14), with one heterozygous. Consanguinity was reported in 11/14 (78.6%) cases, and intrauterine growth restriction (IUGR) was present in all cases. Most mutations were classified as pathogenic (P) or likely pathogenic (LP), with three variants of uncertain significance (VUS) noted.

**Table 1:** Clinical and genetic characteristics of study subjects (n=14).

ID	Family	Sex	Current age (yr)	Age at diagnosis (yr)	Gene	Exon: mutation [transcript]	Variant effect	Classification	Zygosity	Family history	Consanguinity	IUGR
1	Fam-1	F	39	20	*CUL7*	Exon 15: c.2988G>A (p.Trp996Ter) [NM_014780.5]	Nonsense	P	Homo	No	Yes	Yes
2	Fam-2	M	9	1.5	*CUL7*	Intron 16: c.3173-1G>C [NM_014780.5]	Splice site	P	Homo	Yes	No	Yes
3	Fam-3	F	7	Intrauterine	*CUL7*	Intron 17: c.3607 + 1G>C [NM_001168370.1]	Splice site	LP	Homo	Yes	Yes	Yes
4	Fam-3	F	12	3	*CUL7*	Intron 17: c.3607 + 1G>C [NM_001168370.1]	Splice site	LP	Homo	Yes	Yes	Yes
5	Fam-4	F	16	12	*CUL7*	Exon 3: c.784C>T (p.Leu262Phe) [NM_001374872.1]	Missense	VUS	Homo	Yes	Yes	Yes
6	Fam-4	F	15	11	*CUL7*	Exon 3: c.784C>T (p.Leu262Phe) [NM_001374872.1]	Missense	VUS	Homo	Yes	Yes	Yes
7	Fam-5	F	6	1.5	*CCDC8*	Exon 1: c.963del (p.Ala323ProfsTer156) [NM_032040.5]	Frameshift	LP	Homo	Yes	No	Yes
8	Fam-6	F	5	3.5	*CUL7*	ND	–	–	Homo	No	Yes	Yes
9	Fam-7	F	9	0.5	*OBSL1*	Exon 5: c.1997_2049del (p.Gly666AlafsTer56) [NM_015311.3]	Frameshift	P	Homo	Yes	Yes	Yes
10	Fam-7	F	16	1	*OBSL1*	Exon 5: c.1997_2049del (p.Gly666AlafsTer56) [NM_015311.3]	Frameshift	P	Homo	Yes	Yes	Yes
11	Fam-8*	F	11	10	*CCDC8*	Exon 1: c.324_331del (p.Ser108ArgfsTer37) [NM_032040.5]	Frameshift	LP	Homo	Yes	Yes	Yes
12	Fam-9	M	4	2	*OBSL1*	Exon 7: c.2497C>T (p.Arg833Ter) [NM_015311.3]	Nonsense	P	Homo	No	No	Yes
13	Fam-10	F	8	7	*OBSL1*	Exon 14: c.4453C>T (p.Arg1485Ter) [NM_015311.3]	Nonsense	LP	Homo	Yes	Yes	Yes
14	Fam-11**	M	10	3	*OBSL1*	Exon 14: c.4596G>T (p.Arg1532Ser) [NM_015311.3]	Missense	VUS	Hetero	No	Yes	Yes

P; Pathogenic; LP, Likely Pathogenic; VUS, Variant of Uncertain Significance; Homo; Homozygous; Hetero; Heterozygous; IUGR, Intrauterine Growth Restriction; ND, Not documented.

*Case 11 also carries a heterozygous variant in *ACTN2*: c.877-2A>G (LP).

**Case 14 also carries a homozygous variant in *UFSP2*: c.1333G>A (p.Gly445Arg).

At baseline, patients had significantly reduced height with a mean SDS of -3.9. Short stature with dysmorphic/skeletal features was universal, with frequent orthopedic issues (e.g., scoliosis, skeletal dysplasia) and occasional renal involvement (hydronephrosis, nephrotic-range proteinuria). Bone age was usually age-appropriate when assessed; one child had mildly advanced bone age, and one had very low BMD (Z -4.2). IGF-1 was within/low-normal in most tested cases, with two elevated results (**Table 2****).**

**Table 2:** Presentations, growth data, and management outcomes.

ID	Clinical features	MPH (cm)	Baseline height (cm; GV, cm/year; SDS)	Last/final height (cm; GV, cm/year; SDS)	Bone age**	IGF-1 (ng/mL; reference range)	GH therapy (start age; duration; dose)	GH benefit
1	Short stature, dolichocephaly, distinctive facial features, slender fingers, congenital scoliosis, dysplastic hips	172	ND	105* (NA; −8.4)	ND	ND	No—late presentation	NA
2	Short stature, dolichocephaly, distinctive facial features, macrocephaly, left grade 3 hydronephrosis	164	68 (7; −4.2)	114.5 (5; −3.77)	Age appropriate	210 (ref 85–249)	Yes (8 y; 24 mo; 0.75 mg/day)	No—stopped due to headache
3	Short stature, dysmorphic features, prominent heels, hyperextensible joints, nephrocalcinosis, nephrotic syndrome	159	82.8 (4.9; −3.75)	111.3 (6.6; −3.61)	Mildly advanced	615 (ref 87–399; high)	Yes (5 y; 48 mo; 0.6 mg/day)	Yes
4	Short stature, nephrotic-range proteinuria	159	83 (4.3; −6.78)	112.2 (7.9; −4.44)	Age appropriate	228 (ref 188–510)	Yes (6 y; 72 mo; 0.8 mg/day)	Yes
5	Short stature, thin build, speech delay, no dysmorphic or skeletal features	155	127 (6.7; −3.32)	142.5* (1.9; −3.07)	Age appropriate	375 (ref 188–510)	Completed (12 y; 27 mo; 1.33 mg/day)	Yes
6	Short stature, distinctive facial features, triangular face, recurrent UTIs	155	142 (7.24; −1.76)	153.6* (3.11; −1.28)	Age appropriate	326 (ref 268–471)	Yes (12 y; 60 mo; 1.3 mg/day)	Unclear—poor adherence
7	Short stature, low bone mineral density, scoliosis (corrective surgery)	149	66 (5.9; −6.63)	89.3 (6.6; −6.13)	ND; BMD Z−score −4.2 (below expected for age)	101 (ref 80–244)	Yes (6 y; 9 mo; 0.45 mg/day)	Unclear—recently started
8	Short stature, distinctive facial features, triangular face, flat profile, maxillary hypoplasia, dental crowding, developmental and speech delay	163	74 (2.41; −6.18)	86.5 (1.47; −5.75)	ND	42 (ref 34–172)	Yes (5 y; 13 mo; 0.3 mg/day); headache after 1 mo—GH stopped, then resumed; no current side effects	Unclear
9	Short stature, growth failure, osteopenia, deafness	163	70 (9.9; −4.57)	76 (5; −4.3)	ND	195 (ref 34–172; high)	Yes (2 y; 24 mo; 0.33 mg/day)	Yes
10	Short stature, dysmorphic features, skeletal dysplasia, radial head deformity, elbow restriction, brachydactyly	ND	75 (7; −3.95)	144.4* (2.3; −2.85)	ND	219 (ref 87–399)	Completed (8 y; 72 mo; 5 mg/1.5 mL SC, 4 times/week)	Yes
11	Short stature, dysmorphic features, scoliosis	ND	119 (ND; −3.19)	ND	ND	ND	Discontinued after puberty (10 y; 12 mo; dose unknown)	Unclear
12	Short stature, skeletal deformities, distinctive facial features, small head, triangular face, retrognathia, pectus carinatum	ND	71.5 (5.13; −4.38)	81 (6.46; −4.98)	ND	ND	Not yet started	NA
13	FTT, micrognathia, speech delay, musculoskeletal pain, atopy, growth delay, poor school performance, joint pain, arthritis	ND	116.5 (7.22; −1.07)	122.5 (6.96; −0.85)	ND	ND	Not clinically indicated (height SDS −0.85; weight SDS −3.03)	NA
14	Short stature, skeletal deformities, hyperlordosis, osteopenia	ND	83 (6.51; −4.1)	115 (5.31; −3.73)	ND	ND	No—currently followed by orthopedics for post−op implant removal	NA

BMD, bone mineral density; FTT, failure to thrive; GH, growth hormone; GV, growth velocity; IGF-1, insulin-like growth factor 1; MPH, mid-parental height; NA, not applicable; ND, not documented; post-op, postoperative; SC, subcutaneous; SDS, standard deviation score; UTI, urinary tract infection; y, years; mo, months; ref, reference range.

*Indicates patients who have reached final height (FH).

** “Age appropriate” indicates bone age concordant with chronological age.

GH therapy was initiated in 10/14 (71.4%): five showed a clear clinical benefit, one stopped due to headaches (no benefit), and four had indeterminate benefit (recent start/poor adherence/insufficient data). Among patients with paired height SDS, most improved over time (median ΔSDS ≈ +0.43 in GH-treated vs ≈ +0.22 in non-treated; small numbers), with the largest gain of +2.34 SDS in a long-treated child. Four patients had reached final height (including one untreated adult at 105 cm); others remain under follow-up, with GH not started or not indicated in selected cases (**Table 2****).**

**Figure 1:**
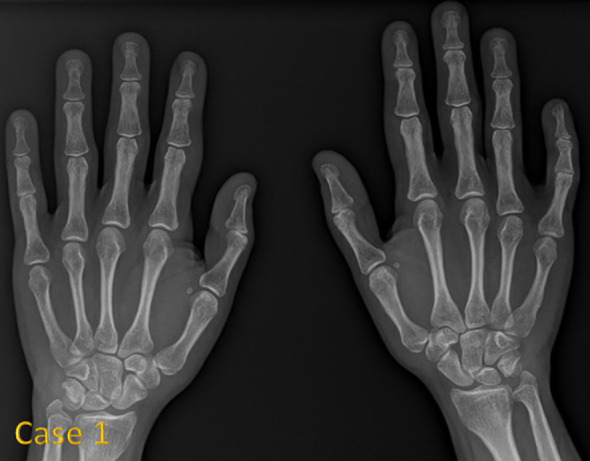
Anteroposterior radiograph of both hands in case 1 reveals slender tubular phalanges.

**Figure 2:**
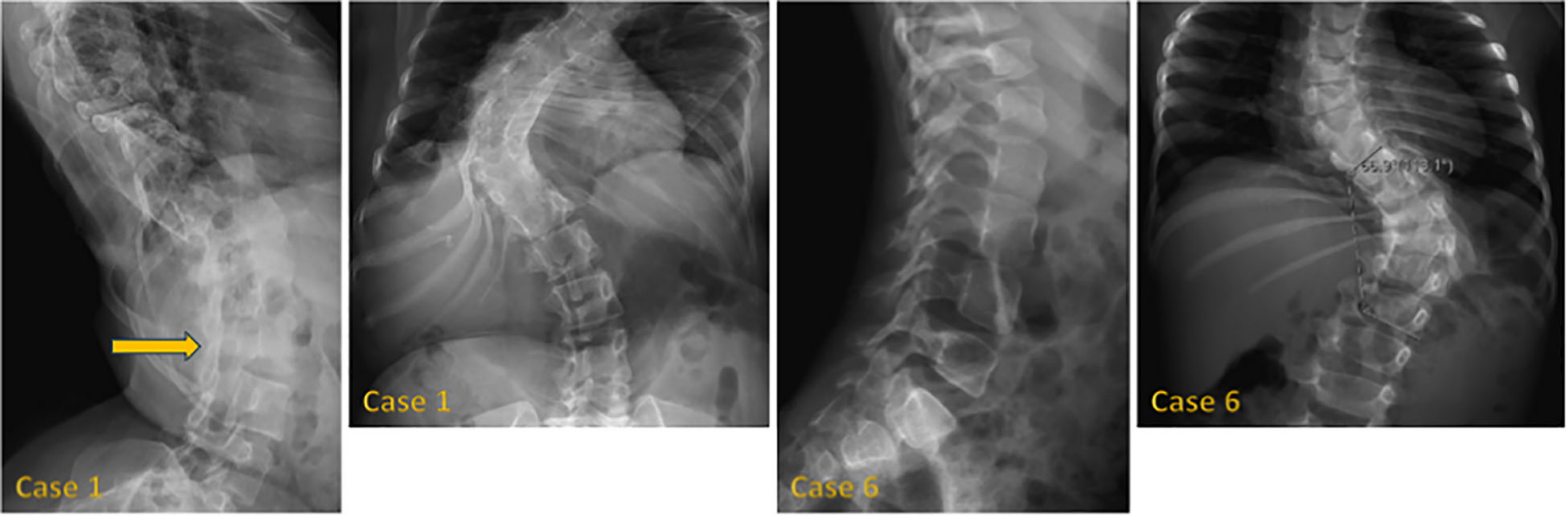
Lateral radiographs of case 1 reveal lumbar hyperlordosis (arrow), while frontal radiographs show thoracolumbar scoliosis and abnormal rib morphology. In case 6, lateral radiographs demonstrate tall lumbar vertebral bodies, and frontal radiographs display thoracolumbar scoliosis.

**Figure 3:**
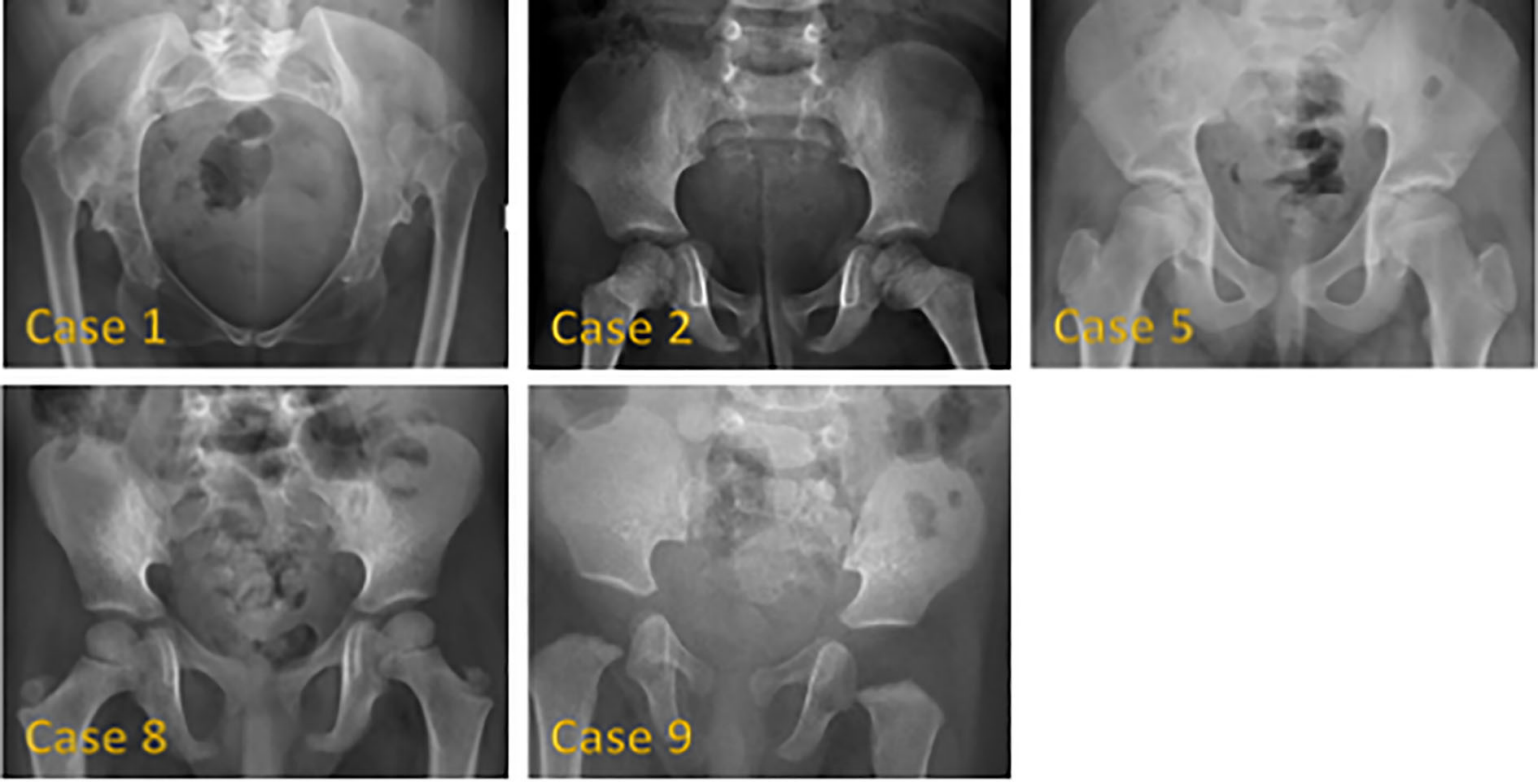
Pelvic radiographs of 5 patients with 3M syndrome. No obvious abnormality was found in Case 2. The pelvic measurements of Cases 5, 8 and 9 are smaller than their peers. Cases 8 and 9 showed a narrow triangular pelvis. Cases 1 and 5 showed a wide pelvis. Case 1 also showed severely dysplastic hip joints, pseudoacetabili formation, and superiorly displaced bilateral femoral heads.

**Figure 4:**
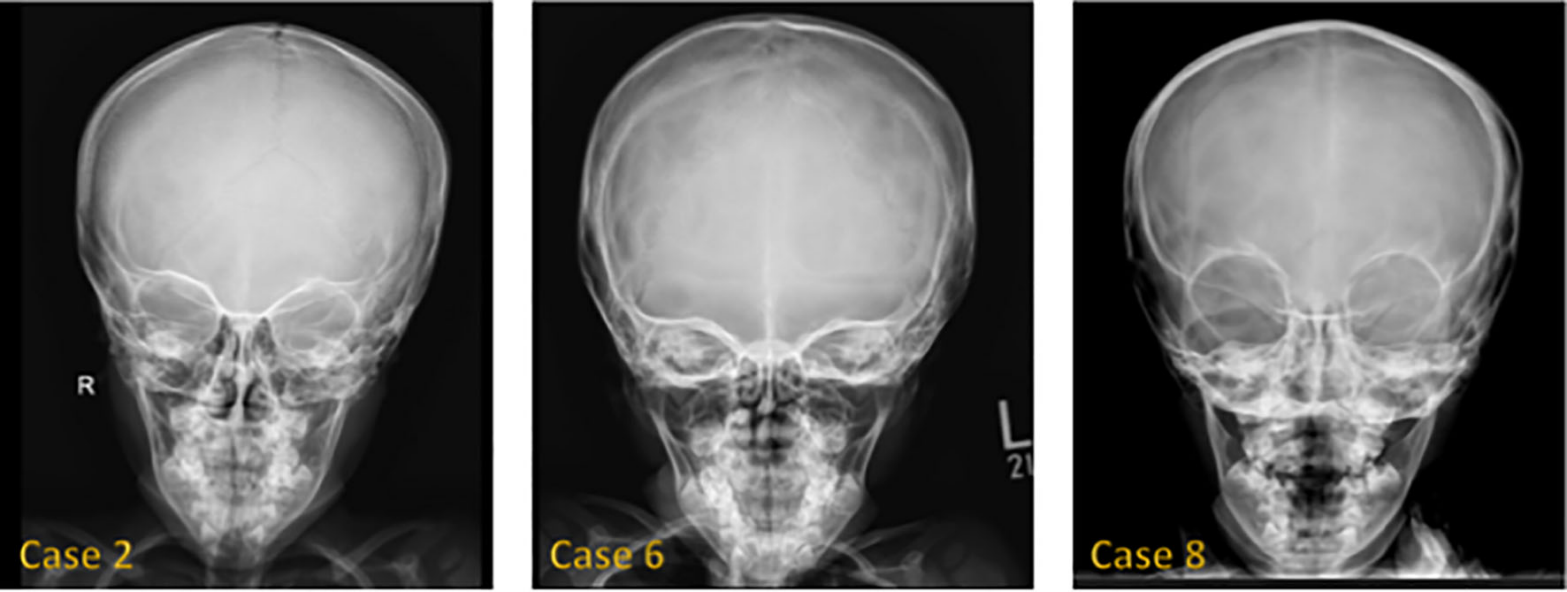
Frontal face radiograph showed a triangular configuration of the face in Cases 2, 6, and 8. Case 8 also showed maxillary hypoplasia, teeth malocclusion, and teeth crowding.

**Figure 1** shows hand radiographs from Case 1 revealing slender tubular phalanges, a characteristic skeletal feature of 3MS. **Figure 2** includes spinal imaging from Cases 1 and 6. Case 1 exhibits lumbar hyperlordosis, thoracolumbar scoliosis, and rib abnormalities, while Case 6 shows tall lumbar vertebral bodies and scoliosis, further supporting skeletal involvement. **Figure 3** illustrates pelvic radiographs from multiple cases. Cases 8 and 9 exhibit narrow triangular pelvis, while Case 1 demonstrates severe hip dysplasia with pseudoacetabuli and displaced femoral heads, reflecting the heterogeneity in pelvic morphology among affected patients. **Figure 4** shows frontal facial radiographs. A triangular face was noted in Cases 2, 6, and 8, with additional craniofacial anomalies in Case 8, including maxillary hypoplasia and dental crowding, features consistent with 3MS-related dysmorphism.

## 4 Discussion

This case series aims to describe the clinical characteristics of 14 Saudi patients who have 3MS, investigate the impact of growth hormone therapy on their growth. Additionally, it provides a review of the recent literature of this condition (3, 10–15, 18, 21–31).

The clinical manifestations observed in this series align with classical 3MS characteristics (10–13) (**Table 3**). Common features included IUGR, postnatal short stature, and a triangular face, frequently accompanied by dental abnormalities, pectus abnormalities, spinal abnormalities, hyperlordosis, and other skeletal abnormalities, such as scoliosis, lordosis, and slender tubular phalanges. One patient (Case 6) was described with recurrent infections, a feature previously documented in Turkey by Ceylan et al. (2023) in a patient with severe combined immunodeficiency (SCID) and 3MS (14). Less frequently described abnormalities, such as macrocephaly, acanthosis nigricans, voracious appetite, and obstructive sleep apnea, were also reported by Yang & Patni (2020) in a patient with a *CUL7* mutation (15).

**Table 3:** Summary of case studies on 3M syndrome (2019–2025): clinical and genetic findings from 20 studies (n = 129 patients).

Author(s), year	Country	Study design	Size	M/F	Positive gene(s)	Clinical presentations
1-Index study (2025)	Saudi Arabia	Case Series	14	3/11	*CUL7 (n=7)* *OBSL1 (n=5)* *CCDC8* (n=2)	Short stature, IUGR, distinctive facial abnormalities (triangular face, micrognathia, dental abnormalities, flat nasal profile), skeletal abnormalities (scoliosis, hyperlordosis, radial abnormalities, pectus deformities), developmental delay, speech delay, low bone mineral density, joint abnormalities, dental crowding, microcephaly, urologic abnormalities (hydronephrosis), renal involvement (2 cases)
2-Akalın et al. (2025) (12)	Turkey	Cohort Study	25	16/9	*CUL7 (n=13)* *OBSL1 (n=11)* *CCDC8* (n=1)	Short stature, IUGR, triangular face, frontal bossing, periorbital fullness, fleshy nasal tip, short nasal bridge, hyperlordosis, joint abnormalities, pes planus, prominently projecting heels, dental abnormalities, aorta abnormalities, Gradenigo’s syndrome in 1 patient.
3-Elsayed et al. (2025) (13)	Egypt	Case Series	11	8/3	*CUL7 (n=8)* *OBSL1 (n=3)*	Short stature, IUGR, triangular face, macrocephaly, square shoulders, short broad thorax, fleshy heels, pes planus, hip dislocation, talipes equinovarus, and variable dysmorphic features (e.g., frontal bossing, pointed chin).
4-Alkhawaldeh et al. (2024) (11)	Jordan	Case Report	1	1/0	*CUL7*	Short stature, distinct facial features, IUGR, normal mental development
5-Piao et al. (2024) (22)	China	Case Report	1	1/0	*OBSL1*	Short stature, IUGR, pronounced forehead, flat nasal bridge
6-Luo et al. (2024) (23)	China	Case Report	1	0/1	*OBSL1*	Square shoulders, scoliosis, long slender tubular bones, no facial dysmorphism
7-Gomez et al. (2024) (24)	Colombia	Case Report	1	0/1	*CUL7*	Short stature, IUGR, prominent forehead, triangular face, bulbous nose, thick lips
8-Wang et al. (2024) (21)	China	Case Report	1	Fetus	*CUL7*	Growth abnormalities *in utero*, including short femur, small abdominal circumference, low fetal weight
9-Xu et al. (2023) (25)	China	Case Series	4	2/2	*CUL7* (n=2), *OBSL1* (n=2)	Short stature, enlarged head circumference, triangular face, low nasal bridge, normal intelligence
10-Ceylan et al. (2023) (14)	Turkey	Case Report	1	1/0	*OBSL1*, *DCLRE1C* (due to SCID)	Recurrent infections, facial dysmorphism, hypotonia, developmental delay, SCID
11-Küçükali et al. (2023) (26)	Turkey	Case Series	8	3/5	*OBSL1*	Short stature, delayed bone age, small for gestational age, triangular face, frontal bossing, short fleshy nose, full lower lip, rib groove, lordosis
12-Akalın et al. (2022) (27)	Turkey	Case Report	1	1/0	*CUL7*	Short stature, prenatal onset, triangular face, macrocephaly, frontal bossing, pectus excavatum
13-Khachnaoui-Zaafrane et al. (2022) (28)	Tunisia	Case Series	7	3/4	*CUL7*	Facial dysmorphism, skeletal abnormalities (lumbar lordosis, hyperextensible joints), spina bifida occulta, single transverse palmar creases
14-Tüysüz et al. (2021) (18)	Turkey	Cohort Study	19	10/9	*CUL7* (n=11), *OBSL1* (n=8)	Triangular face, short fleshy nose, full lower lip, rib groove, lordosis, slender long bones, facial infantile hemangioma
15-Isik et al. (2021) (3)	Turkey	Case Series	4	2/2	*CUL7* (n=2), *OBSL1* (n=2)	IUGR, macrocephaly, typical facial features
16-Lee et al. (2020) (29)	Korea	Case Report	2	0/2	*OBSL1*	Short stature, macrocephaly, frontal bossing, triangular face, prominent philtrum, full lips, short neck, fifth-finger clinodactyly
17-Yang & Patni (2020) (15)	USA	Case Report	1	1/0	*CUL7*	IUGR, macrocephaly, skeletal abnormalities, GH insensitivity, morbid obesity, voracious appetite, acanthosis nigricans, tonsillar hypertrophy, obstructive sleep apnea
18-Simsek‐Kiper et al. (2019) (30)	Turkey	Cohort Study	24	12/12	*CUL7 (n=10)* *OBSL1 (n=8)* *BMP2 (n=2)* NR *(n=4)*	Short stature, short extremities, IUGR, delayed bone age, skeletal abnormalities
19-HabibUllah et al. (2019) (31)	Saudi Arabia	Case Report	1	1/0	*CUL7*	Short stature, low weight, developmental delay, dysmorphic features (large head, triangular face, upturned nostrils, clinodactyly)
20-Shaikh et al. (2019) (10)	India	Case Report	2	1/1	*CUL7*	IUGR, facial dysmorphology, broad thorax, heel protrusion

CUL7, Cullin 7; OBSL1, Obscurin-like 1; SCID, Severe Combined Immunodeficiency; IUGR, Intrauterine Growth Retardation; BMP2, Bone Morphogenetic Protein 2; NR, not reported.

Genetically, we identified variants predominantly in *CUL7*, *OBSL1*, and *CCDC8*, reflecting autosomal recessive inheritance, a pattern frequently described in 3MS cases (**Table 3**). The most frequently affected gene was *CUL7*, followed by *OBSL1*, while *CCDC8* variants were rare, which is consistent with the findings by Akalın et al. (2025) (12). Consanguinity was present in nearly 79% of cases, which may explain the predominantly homozygous variants in this population. *CUL7* mutations identified in cases 1 and 2 — c.2988G>A (p.Trp996Ter) and c.3173-1G>C, respectively — have previously been described in 3MS cases (16, 17).

In mechanistic context, the three canonical 3MS genes form an interrelated “3M complex” that participates in cytoskeletal integrity, mitotic progression, and ubiquitin–proteasome–mediated protein turnover (2, 9, 12, 13, 20). *CUL7* encodes a scaffold of a cullin-RING E3 ubiquitin ligase that, together with adaptor proteins, regulates turnover of signaling intermediates, including components of insulin/IGF pathways (2, 7, 9, 20). Experimental work indicates that perturbations of CUL7 can dysregulate IRS-1 handling and downstream PI3K–AKT signaling, contributing to impaired chondrocyte proliferation and skeletal growth despite ostensibly intact upstream GH stimulation (2, 9, 13, 25). *OBSL1* (a cytoskeletal adaptor) and *CCDC8* (a coiled-coil protein with roles in cell division and genome surveillance) interact with *CUL7*; loss of function across any of these partners disrupts the complex, with convergent effects on growth-factor signaling and endochondral ossification (2, 9, 13, 20). Clinically, this biology predicts GH insensitivity or post-receptor resistance, a pattern we observed—several children had normal or even elevated IGF-1 while linear growth remained suboptimal (18, 19, 26, 29).

As for management, of the ten reported cases where GH therapy was administered, the response was highly variable. Five cases demonstrated a notable improvement, while the other five showed no clear response or discontinued therapy due to factors like puberty or poor compliance. This variability is consistent with previous reports; for instance, Tüysüz et al. (2021) described analogous cases where GH treatment improved growth outcomes in only a subset of children with 3MS (18). Similarly, Altun et al. (2025) observed modest but statistically significant improvements in annual growth velocity (from 5.3 to 6.1 cm/year) in eight pediatric cases, despite evidence of underlying GH resistance indicated by elevated IGF-1 levels in some patients (19). While final height in these studies remained significantly below average, the stabilization or slight improvement in height SDS suggests a potential, albeit limited, benefit from GH therapy (19).

The challenges of this treatment are further highlighted across other studies. In one study, three out of four children exhibited significant growth acceleration with recombinant human GH (rhGH), though their long-term outcomes remain under observation (32). Another study reported that five out of seven patients discontinued GH treatment due to an insufficient growth response, underscoring the difficulty in sustaining effective therapy over time (Küçükali et al., 2023) (26). The genetic background of 3MS, particularly mutations in the CUL7 and OBSL1 genes, is thought to influence this variable response and contribute to the observed GH resistance (Xu et al., 2023) (25). In cases of suspected resistance, recombinant human IGF-1 (rhIGF-1) has been trialed as an alternative; however, one such report noted it did not provide substantial height benefits and was associated with side effects like obesity and acanthosis (Yang & Patni, 2020) (15). To potentially enhance outcomes, combination therapies have been explored, with one report of two siblings showing height gains following a regimen of GH and a gonadotropin-releasing hormone (GnRH) agonist (Lee et al., 2020) (29).

Beyond pharmacological interventions, the comprehensive management of 3MS includes surgical interventions for skeletal and joint abnormalities, as well as adaptive measures for persistent short stature. Furthermore, genetic counseling is recommended for affected families to explain the autosomal recessive inheritance pattern, discuss the 25% recurrence risk, and review options such as preimplantation genetic testing and prenatal ultrasound for early diagnosis (20, 21).

This study has a few limitations. Since it included only a small number of patients, the results may not apply to all individuals with 3MS. Also, because the data were collected by looking back at medical records, there’s a chance some details were missed or not recorded consistently. Finally, the study took place at just one specialized hospital, so the findings might not represent how 3MS is diagnosed or treated in other hospitals or regions.

In conclusion, this study highlights the clinical variability of 3MS, predominantly homozygous variants in *CUL7*, *OBSL1*, and *CCDC8*, and the potential role of GH therapy in improving growth outcomes in some cases. Nevertheless, the response to treatment is heterogeneous and underscores the necessity for individualized management plans and ongoing follow-up. Furthermore, genomic testing and proper genetic counseling are crucial for guiding future family planning and disease management.
